# Life

**Published:** 1891-11

**Authors:** 


### LIFE.

Life ! we've been long together,
Through pleasant and through cloudy weather ;
'Tis hard to part when friends are dear ;
Perhaps 'twill cost a sigh, a tear ;
Then steal away, give little warning,
Choose thine own time,
Say not "Good Night," but in some brighter clime
Bid me "Good Morning."

